# An Outcome Study of Anterior Cervical Discectomy and Fusion among Iranian Population

**DOI:** 10.1155/2016/4654109

**Published:** 2016-08-18

**Authors:** Ali Haghnegahdar, Mahsa Sedighi

**Affiliations:** Department of Neurosurgery, Neurospine Section, Chamran Hospital, Shiraz Medical College, Shiraz University of Medical Sciences, Shiraz 71937-11351, Iran

## Abstract

*Background and Aim*. First-line treatment strategy for managing cervical disc herniation is conservative measures. In some cases, surgery is indicated either due to signs/symptoms of severe/progressive neurological deficits, or because of persistence of radicular pain despite 12 weeks of conservative treatment. Success for treatment of cervical disc herniation using ACDF has been successfully reported in the literature. We aim to determine the outcome of ACDF in treatment of cervical disc herniation among Iranians.* Methods and Materials/Patients*. In a retrospective cohort study, we evaluated 68 patients who had undergone ACDF for cervical disc herniation from March 2006 to March 2011. Outcome tools were as follows: (1) study-designed questionnaire that addressed residual and/or new complaints and subjective satisfaction with the operation; (2) recent (one week prior to the interview) postoperative VAS for neck and upper extremity radicular pain; (3) Japanese Orthopaedic Association Myelopathy Evaluation Questionnaire (JOACMEQ) (standard Persian version); and (4) follow-up cervical Magnetic Resonance Imaging (MRI) and lateral X-ray.* Results*. With mean follow-up time of 52.93 (months) ± 31.89 SD (range: 13–131 months), we had success rates with regard to ΔVAS for neck and radicular pain of 88.2% and 89.7%, respectively. Except QOL functional score of JOAMEQ, 100% success rate for the other 4 functional scores of JOAMEQ was achieved.* Conclusion*. ACDF is a successful surgical technique for the management of cervical disc herniation among Iranian population.

## 1. Introduction

Cervical radiculopathy could be caused by disc herniation, spondylosis, instability, and trauma or on rare occasions by tumors [1]. Majority of cervical radiculopathies are caused by cervical spondylosis, while 25% are the result of disc herniation [2]. Cervical radiculopathy point prevalence and annual incidence have been reported at a population of 3.5/1,000 and 83/100,000, respectively [3]. Cervical disc herniation mostly affects individuals aged between 30 and 50 years [4]. C5-C6 level is the most common involved level of herniation [5]. First-line treatment in management of CDH is conservative measures. Approximately 83% of patients with cervical radiculopathy respond to conservative treatment methods [5], while an approximate one-third of patients will suffer from persistent symptoms [6]. Surgery is indicated for cases that have signs/symptoms of severe/progressive neurological deficits and persistence of radicular pain despite 12 weeks of conservative treatment. Surgery is mostly performed via an anterior approach with or without fusion [7], although traditionally posterior approach is another method [3]. Outcome of surgical management for cervical disc herniation has shown a success rate of 66 to 98% [8]. ACDF has demonstrated good results in terms of pain relief and patient satisfaction [9]. Its success rate has been reported to achieve good or excellent outcome in 93% [10].

## 2. Materials and Methods

From April 2003 to June 2014, 1280 cases of spine surgeries were performed by the senior author at a single centre. Surgical indications for cervical disc herniation (CDH) were (1) progressive myelopathy; (2) persistence or worsening of radiculopathy despite 12 weeks of medical treatment; and (3) motor deficit or intractable pain. Our inclusion criteria were (1) single- or multilevel CDH and (2) more than 12 months of postoperative follow-up. Cases were excluded due to (1) coexistent spine pathologies; (2) history of previous spine surgery; and (3) less than 12 months of postoperative follow-up.

Sixty-eight cases of CDH that were managed by ACDF were included in this series. The outcome instruments were (1) study-designed questionnaire that addressed residual and/or new complaints and subjective satisfaction with the operation; (2) recent (one week prior to the interview) postoperative VAS for neck and upper extremity radicular pain; (3) Japanese Orthopaedic Association Myelopathy Evaluation Questionnaire (JOACMEQ) (standard Persian version); and (4) follow-up cervical Magnetic Resonance Imaging (MRI) and lateral X-ray.

Preoperative medical information which consisted of preoperative symptoms, duration of pain (from onset up to surgery), physical examination, and pain severity using Visual Analogue Scale (VAS) was recorded at the time of operation by the senior author. The operation notes were reviewed for intraoperative complications. The follow-up notes were read for the postoperative course. Our study population was contacted by phone and informed about the research project and invited for a follow-up visit. A physician working in the field of spine research carried out the follow-up visits.

This is the first study that addresses the long-term clinical and radiographic outcome and influential factors among Iranian patients who underwent ACDF for herniated cervical disc using polyetheretherketone (PEEK) cage stand-alone technique.

## 3. Data Analysis

Data analysis was performed with SPSS version 16.0 (SPSS, Inc., Chicago, IL, USA). Statistical significance was set at level of 0.05. For descriptive statistics, central and dispersion tendencies were performed. For comparison between qualitative variables, nonparametric test (chi-square) test was done. Comparing qualitative and quantitative variables was analysed by using nonparametric test (Mann-Whitney *U* test).

## 4. Results

Our mean follow-up time was 52.93 (months) ± 31.89 SD (range: 13–131 months). 68 cases (99 segments) were studied. 42 cases had one-level disc herniation and 21 and 5 patients were operated on for two- and three-level involvements, respectively. 34 were men and 34 were female. Study population mean age was 47.26 ± 11.138 SD. Majority (39, 57.4%) of cases had a sedentary job. Most were nonsmokers, 64 (94.1%). Mean preoperative VAS (one week prior to operation) for neck pain and radicular pain were 9.32 ± 2.40 SD and 9.29 ± 2.41 SD, respectively. Mean postoperative VAS (at the time of follow-up) for neck and radicular pain were 1.28 ± 2.50 SD and 1.03 ± 2.22 SD. Most (22, 32.4%) of our cases had disc herniation at the level of C5-C6. Two-level disc herniation was mostly (10, 47.6%) encountered at C5-C6/C6-C7. Other patient data is presented in Table 1. Fusion was confirmed with imaging studies in all 42 cases that came for follow-up imaging study (100% fusion rate). No statistically significant relation was noted between radiologic and outcome measures. Furthermore, radiologic findings were not shown to affect the severity of residual complaints statistically.

Subjective satisfaction with the surgery was 95.6%. Success rates for clinical outcome with regard to VAS for neck and radicular pain were 88.2% and 89.7%, respectively (mean ΔVAS for neck pain: 8.04 ± 3.27 SD; mean ΔVAS for radicular pain: 8.26 ± 3.10 SD). Success rates for 5 scores of JOAMEQ are shown in Table 2.

We had no intraoperative complications. Early postoperative complications were hoarseness (3 cases, 4.4%), C5 root palsy (one case, 1.5%), and dysphagia (one case, 1.5%). Late postoperative complications were 8 (19%) cases of subsidence, 6 (14.3%) cases of adjacent segment degeneration, 2 (2.94%) cases of adjacent segment disease, 1 (1.5%) case of right upper broken screw, 1 (1.5%) case of screw loosening, and one (1.5%) case of graft extrusion. Among cases who were diagnosed with subsidence, 6 (75%) had undergone single-level ACDF (C5-C6, C6-C7) and 2 had undergone 2-level ACDF.

**Figure 1:**
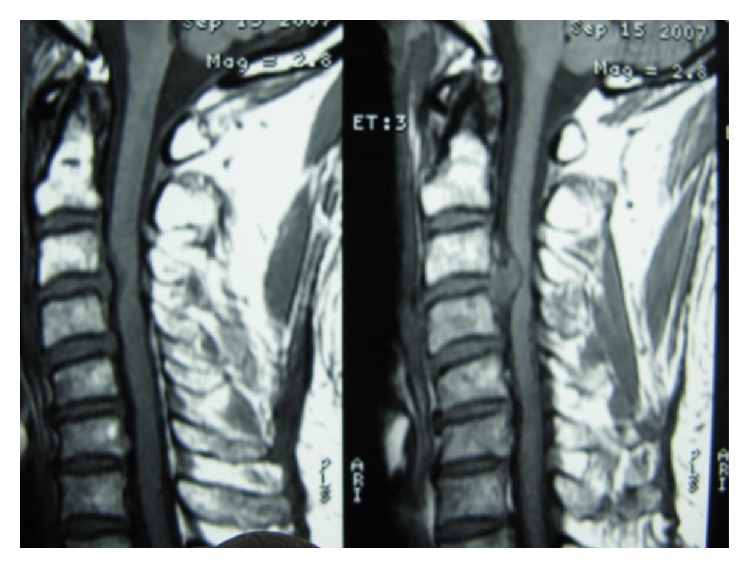
Pre- and postoperative images of single-level ACDF.

**Figure 2 fig2:**
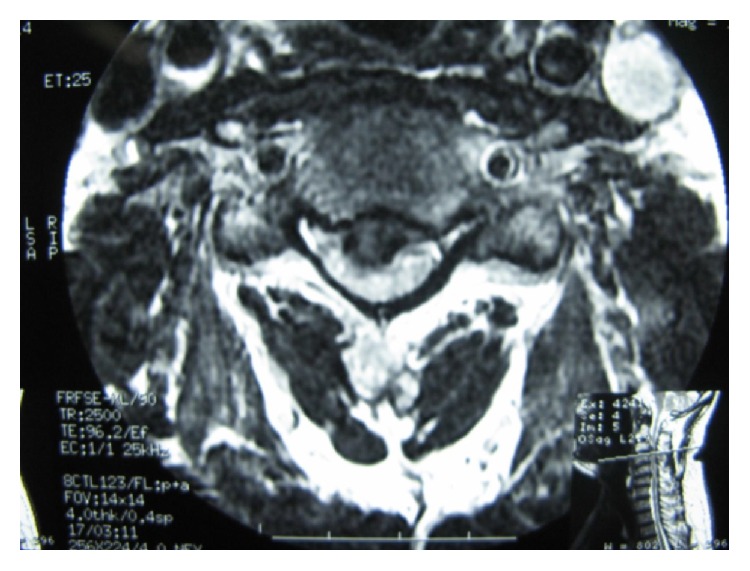
Pre- and postoperative images of single-level ACDF.

**Figure 3 fig3:**
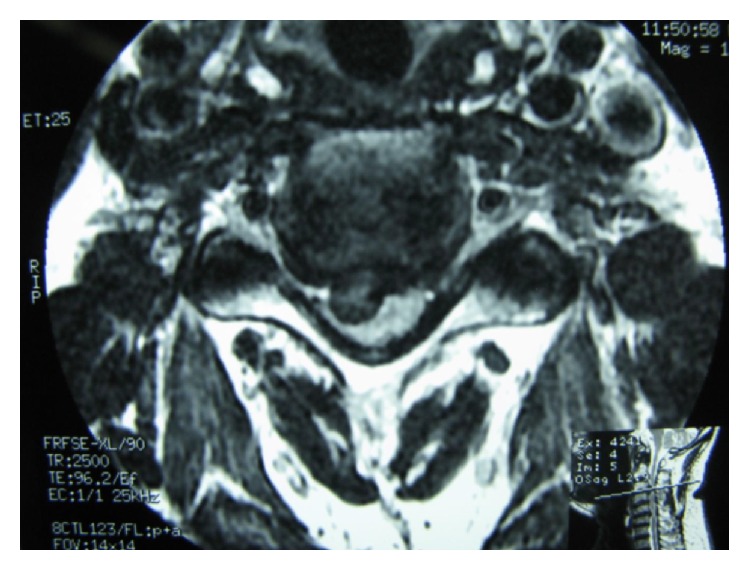
Pre- and postoperative images of single-level ACDF.

**Figure 4 fig4:**
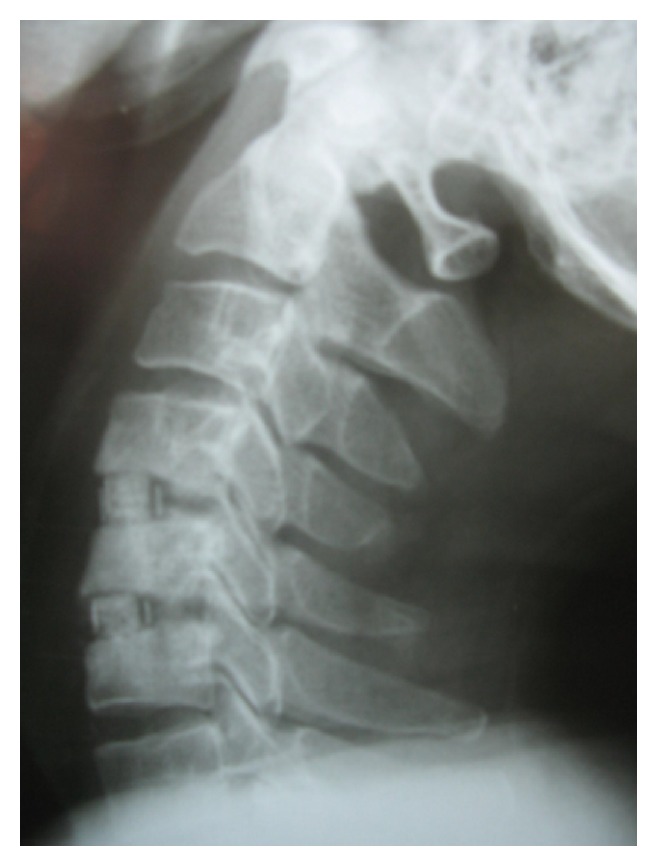
Postoperative X-ray of 2-level ACDF.

**Figure 5 fig5:**
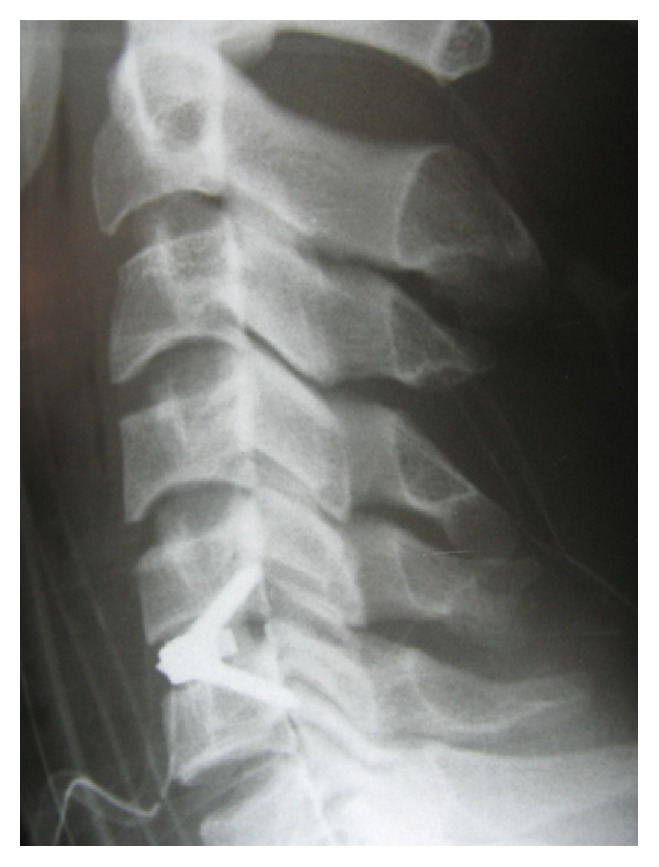
Postoperative X-ray of ZeroP.

**Figure 6:**
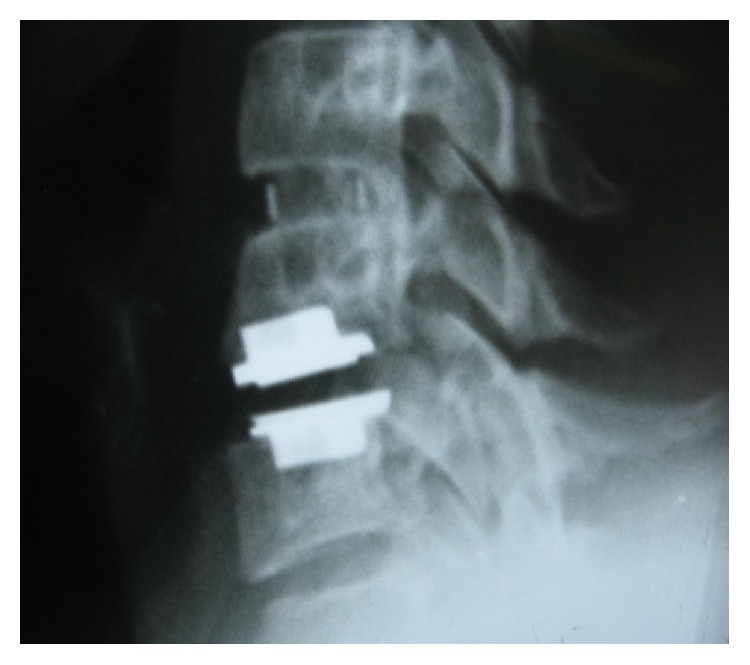
Single-level ACDF with adjacent level disease that underwent operation for implanting an artificial disc.

During the follow-up time, none of the patients complained of symptoms recurrence. 47.1% of patients complained of residual complaints at the time of the follow-up. The most common (7, 10.3%) residual complaint was sensory deficits, followed by cases (6, 8.8%) who still suffered from both upper extremity radicular pain and sensory deficits at the final follow-up. Four (5.8%) reported new complaints. These were neck pain (1 case; cervical MRI showed 10% cage subsidence; VAS: 5), contralateral to preoperative side of radicular pain (1 case; cervical MRI demonstrated different level disc herniation), limitation of shoulder abduction (1 case; diagnosed with C5 root palsy), and sensory deficits (1 case, VAS: 8). Figures 1 [Fig fig2] [Fig fig3] [Fig fig4] [Fig fig5]–6 demonstrate imaging studies for some of our cases.

The outcome of surgery did not differ by type of job, smoking, preoperative neck pain or sensory complaints, preoperative hypoesthesia, duration of pain, or level of disc herniation. The surgical outcome was significantly (*p* value: 0.039) better for men (mean rank: 38.09) in comparison to women (mean rank: 30.91). Older patients had lower scores in UEF (*p* value: 0.043), LEF (*p* value: 0.001), BF (*p* value: 0.001), and QOL (*p* value: 0.01). Presence of preoperative radicular pain affected UEF (*p* value: 0.046) (mean UEF score for those with preoperative radicular pain was 96.69 ± 10.43 SD and for individuals without preoperative radicular pain was 88.30 ± 17.99 SD). Preoperative Hoffmann's sign affected LEF (*p* value: 0.008), BF (*p* value: 0.007), and QOL (*p* value: 0.029). Preoperative Babinski sign had an effect on LEF and BF (*p* value: 0.01).

## 5. Discussion

In this study, we achieved improvement with regard to neck and arm pain of 88.2% and 89.7% of our study population. With an average follow-up period of 18 months, Kwon et al. [11] demonstrated rates of 96.1% and 82.1% for neck and arm pain, respectively, based on VAS. At mean follow-up of 25.6 months, Liu et al. [12] demonstrated significant clinical improvement with regard to VAS for arm and neck pain. Furthermore, Dagli et al. [13] reported a significant decrease in VAS at two-year follow-up. A statistically significant decrease for VAS, both for neck and arm pain, was achieved for study population in Park et al.'s series [14] at mean follow-up period of 12 months. Except QOL functional score, the other four success rates calculated with JOAMEQ ranged between 70.6 and 83.8%.

With regard to residual complaints, we observed that 47.1% of our cases complained of minor residual symptoms at the final follow-up. Peolsson [15] reported that 70% of their study population suffered from persistent pain and disability at 6-year follow-up.

Studies of Bohlman et al. [16], Lied et al. [17], and Gaetani et al. [18] did not show age affecting their outcome. In concordance with other studies [19–21], we showed that men with younger age achieved better outcome. In agreement with study of Bohlman et al. [16], we also could not find a relation between smoking status and outcome.

In our series, duration of preoperative symptoms did not have a statistically significant effect on any of our outcome measures. Omidi-Kashani et al. [22] demonstrated no correlation between duration of symptoms and surgical outcome in their evaluation on cases with cervical spondylotic radiculopathy who were treated with ACDF. Lied et al. [17] also achieved no significant correlation between preoperative duration of pain and pain relief.

ACDF has been advocated as a safe procedure, but complications could still arise. Among its complications are nonunion, postoperative dysphagia [23], recurrent laryngeal nerve palsy, esophageal tear, carotid artery injury, vertebral artery injury, neurologic deficit, postoperative respiratory embarrassment, and disc space infection [24]. Injury to RLN was found by Flynn to be the most frequently encountered neurologic complication [25]. Two studies [23, 26] reported dysphagia as the most common ACDF-related complication. We had one case (1.5%) with dysphagia, which is lower than the incidence of dysphagia reported in other studies that ranged between 2.5 and 21.3% [27–30].

In a study conducted by Chen et al. [31], incidence of 0.16% was reported for hoarseness, while this rate was reported higher in Baron et al.'s [32] series at a rate of 4.9%, which is close to the incidence of 4.4%, which we observed among our study population.

An average rate of 4.3% (range: 1.6%–12.1%) has been documented in literature for the incidence of C5 root palsy after anterior decompression and fusion [33]. In our series, we had one case (1.5%) with C5 root palsy which is lower than the incidence of Kim et al.'s [34] series which reported a rate of 4.3%. Incidence of graft extrusion has ranged between 0 and 0.88% [12, 26, 35]. Prevalence of this complication (1.5%) in our series was higher than other previously reported studies. With an average follow-up duration of 18 months, Kulkarni et al. [36] reported that none of their study population had cage extrusion or migration. Cabraja et al. [37] demonstrated no cage extrusion on average follow-up period of 28.4 months. In a study conducted by Nanda et al. [26], cases with graft extrusion had persistent neurological symptoms after the operation, but graft extrusion in our patient was associated with a new-onset neck pain.

Incidence of adjacent segment degeneration (ASdeg) after ACDF has been reported to range from 16 to 51 [38, 39]. 21.95% of Dagli et al.'s [13] study population were found with ASdeg at 2-year follow-up. Herkowitz et al. [40] study with 4.5-year follow-up showed that 41% of their series developed ASdeg. In our series, we showed a rate of 14.3% after a follow-up period of 4.41 yrs ± 2.67  SD. Some cases of ACDF develop symptomatic adjacent segment disease (ASdx). The reported incidence for ASdx ranges between 2 [38] and 41% [40]. Two cases (2.94%) with symptomatic ASdx were observed in our series. ASdx progression to a degree that requires additional surgery was the case for one of our patients (1.5%). With an average follow-up period of 6 years, Bohlman et al. [16] described that 9% of their patients required additional surgery for ASdx. In another series [41], 17% of the study population required additional surgery for ASdx at an average of 4.5 years of follow-up.

The prevalence of subsidence in our study was 19%, which is higher than Cabraja et al.'s [37] series that reported PEEK cage subsidence in 14.3% of their cases at mean follow-up of 28.4 months. At 2-year follow-up period, Galhom [35] had 3 cases (7.5%) with subsidence. At shorter follow-up periods, rate of PEEK cage associated subsidence of 8.1% has been observed at mean follow-up time of 18.9 months by Ha et al. [42]. And with an average follow-up of 12 months, Park et al. [14] reported that 22.6% of their cases had subsidence. In a study conducted by Song et al. [43] subsidence was observed at rate of 32.3%.

Kao et al. [44] found that subsidence was significantly associated with gender and number of treatment levels and treatment at C5–7. In Kast et al.'s series [45], age and gender did not influence subsidence. We achieved no correlation between age, gender, number of treatment levels, and subsidence; although with no statistically significant difference, the majority (75%) of cases diagnosed with subsidence had undergone single-level ACDF (C5-C6, C6-C7).

Kulkarni et al. [36] reported a rate of 93.33% for PEEK cage fusion at 6 months. At a mean follow-up of 10 months, 100% fusion rate was observed for Cho et al.'s [46] cases. With an average of 18 months of follow-up, Kulkarni et al.'s [36] study population fusion was maintained at their last follow-up. At* mean* follow-up of 28.4 months, Cabraja et al. [37] achieved a fusion rate of 88.1% for PEEK cage. At mean follow-up of 25.6 months, Liu et al. [12] observed fusion rate of 72%. Song et al. [43] had 78.9% fusion. In a prospective study by Niu et al. [47], fusion rate at 12-month follow-up was 100% for PEEK cage group. With mean follow-up period of 18.9 months, Ha et al. [42] achieved 94.5% fusion. We achieved 100% of fusion rate at mean follow-up time close to 53 months.

## 6. Conclusion

ACDF is a successful surgical technique for the management of cervical disc herniation among Iranian population.

## Figures and Tables

**(a):** 

Variable	Frequency (number, percentage)
*Job*	
Sedentary	39, 57.4
With some level of activity	20, 29.4
Heavy	9, 13.2

*Preoperative symptomatology *	
Neck pain	59, 86.8
Upper extremity radicular pain	59, 86.8
Sensory complaints	50, 73.5
Headache	6, 8.8
Incontinency	2, 2.9
Chest discomfort	1, 1.5
Walking disability	11, 16.2
Limb stiffness	5, 7.4

*Pain duration from onset up to surgery *	
<3 months	24, 35.3
3–6 months	16, 23.5
6–12 months	5, 7.4
12–24 months	1, 1.5
>24 months	22, 32.4

**(b):** 

Preoperative signs	Frequency
*Reflexes*	
Normal upper extremity reflex	30, 44.1
Hyperreflexia of upper extremity	25, 36.8
Hyporeflexia of upper extremity	13, 19.1
Normal lower extremity reflex	43, 63.2
Hyperreflexia in lower extremity	22, 32.4
Hyporeflexia in lower extremity	3, 4.4

*Upper extremity muscle power (in one or two dermatomal groups)*	
3/5	2, 2.9
4/5	48, 70.6
5/5	18, 26.5

*Lower extremity muscle power*	
3/5	1, 1.5
4/5	14, 20.6
5/5	53, 77.9

*Hoffmann's sign*	
Positive	25, 36.8
Negative	43, 63.2

*Babinski sign*	
Upward	18, 26.5
Downward	50, 73.5

*Upper extremity hypoesthesia*	
Positive	40, 58.8
Negative	28, 41.2

**Table 2:** JOAMEQ functional classes scores and success rates.

Functional class	Median score	Success rate
CSF	100	77.9
UEF	100	83.8
LEF	100	70.6
BF	100	79.4
QOL	56.25	11.8

CSF: cervical spine function; UEF: upper extremity function; LEF: lower extremity function; BF: bladder function; QOL: quality of life.

## References

[1] Caridi J. M., Pumberger M., Hughes A. P. (2011). Cervical radiculopathy: a review. *HSS Journal*.

[2] Radhakrishnan K., Litchy W. J., O'Fallon W. M., Kurland L. T. (1994). Epidemiology of cervical radiculopathy: a population-based study from Rochester, Minnesota, 1976 through 1990. *Brain*.

[3] Todd A. G. (2011). Cervical spine: degenerative conditions. *Current Reviews in Musculoskeletal Medicine*.

[4] Akhavan-Sigari R., Rohde V., Alaid A. (2013). Cervical spinal canal stenosis and central disc herniation c3/4 in a man with primary complaint of thigh pain. *Journal of Neurological Surgery Reports*.

[5] Clavenna A., Dossett A. B. (2005). Anterior cervical diskectomy and fusion. *Operative Techniques in Sports Medicine*.

[6] Lees F., Turner J. W. (1963). Natural history and prognosis of cervical spondylosis. *British Medical Journal*.

[7] Saal J. S., Saal J. A., Yurth E. F. (1996). Nonoperative management of herniated cervical intervertebral disc with radiculopathy. *Spine*.

[8] Alrawi M. F., Khalil N. M., Mitchell P., Hughes S. P. (2007). The value of neurophysiological and imaging studies in predicting outcome in the surgical treatment of cervical radiculopathy. *European Spine Journal*.

[9] Engquist M., Löfgren H., Öberg B. (2013). Surgery versus nonsurgical treatment of cervical radiculopathy: a prospective, randomized study comparing surgery plus physiotherapy with physiotherapy alone with a 2-year follow-up. *Spine*.

[10] Klein G. R., Vaccaro A. R., Albert T. J. (2000). Health outcome assessment before and after anterior cervical discectomy and fusion for radiculopathy: a prospective analysis. *Spine*.

[11] Kwon B., Kim D. H., Marvin A., Jenis L. G. (2005). Outcomes following anterior cervical discectomy and fusion: the role of interbody disc height, angulation, and spinous process distance. *Journal of Spinal Disorders & Techniques*.

[12] Liu H., Ploumis A., Li C., Yi X., Li H. (2012). Polyetheretherketone cages alone with allograft for three-level anterior cervical fusion. *ISRN Neurology*.

[13] Dagli M., Er U., Şimşek S., Bavbek M. (2013). Late results of anterior cervical discectomy and fusion with interbody cages. *Asian Spine Journal*.

[14] Park J.-I., Cho D.-C., Kim K.-T., Sung J.-K. (2013). Anterior cervical discectomy and fusion using a stand-alone polyetheretherketone cage packed with local autobone: assessment of bone fusion and subsidence. *Journal of Korean Neurosurgical Society*.

[15] Peolsson A. (2007). Investigation of clinically important benefit of anterior cervical decompression and fusion. *European Spine Journal*.

[16] Bohlman H. H., Emery S. E., Goodfellow D. B., Jones P. K. (1993). Robinson anterior cervical discectomy and arthrodesis for cervical radiculopathy. Long-term follow-up of one hundred and twenty-two patients. *The Journal of Bone and Joint Surgery—Series A*.

[17] Lied B., Roenning P. A., Sundseth J., Helseth E. (2010). Anterior cervical discectomy with fusion in patients with cervical disc degeneration: a prospective outcome study of 258 patients (181 fused with autologous bone graft and 77 fused with a PEEK cage). *BMC Surgery*.

[18] Gaetani P., Tancioni F., Spanu G., Rodriguez Baena Y. R. (1995). Anterior cervical discectomy: an analysis on clinical long-term results in 153 cases. *Journal of Neurosurgical Sciences*.

[19] Bertalanffy H., Eggert H.-R. (1988). Clinical long-term results of anterior discectomy without fusion for treatment of cervical radiculopathy and myelopathy. A follow-up of 164 cases. *Acta Neurochirurgica*.

[20] Hamburger C., Festenberg F. V., Uhl E. (2001). Ventral discectomy with PMMA interbody fusion for cervical disc disease: long-term results in 249 patients. *Spine*.

[21] Eriksen E. F., Buhl M., Fode K. (1984). Treatment of cervical disc disease using Cloward's technique the prognostic value of clinical preoperative data in 1,106 patients. *Acta Neurochirurgica*.

[22] Omidi-Kashani F., Ghayem Hasankhani E., Ghandehari R. (2014). Impact of age and duration of symptoms on surgical outcome of single-level microscopic anterior cervical discectomy and fusion in the patients with cervical spondylotic radiculopathy. *Neuroscience Journal*.

[23] Fountas K. N., Kapsalaki E. Z., Nikolakakos L. G. (2007). Anterior cervical discectomy and fusion associated complications. *Spine*.

[24] Zdeblick T. A. (1993). Anterior cervical discectomy and fusion. *Operative Techniques in Orthopaedics*.

[25] Flynn T. B. (1982). Neurologic complications of anterior cervical interbody fusion. *Spine*.

[26] Nanda A., Sharma M., Sonig A., Ambekar S., Bollam P. (2014). Surgical complications of anterior cervical diskectomy and fusion for cervical degenerative disk disease: a single surgeon's experience of 1576 patients. *World Neurosurgery*.

[27] Singh K., Marquez-Lara A., Nandyala S. V., Patel A. A., Fineberg S. J. (2013). Incidence and risk factors for dysphagia after anterior cervical fusion. *Spine*.

[28] Bazaz R., Lee M. J., Yoo J. U. (2002). Incidence of dysphagia after anterior cervical spine surgery: a prospective study. *Spine*.

[29] Lee M. J., Bazaz R., Furey C. G., Yoo J. (2007). Risk factors for dysphagia after anterior cervical spine surgery: a two-year prospective cohort study. *The Spine Journal*.

[30] Riley L. H., Skolasky R. L., Albert T. J., Vaccaro A. R., Heller J. G. (2005). Dysphagia after anterior cervical decompression and fusion: prevalence and risk factors from a longitudinal cohort study. *Spine*.

[31] Chen C.-C., Huang Y.-C., Lee S.-T., Chen J.-F., Wu C.-T., Tu P.-H. (2014). Long-term result of vocal cord paralysis after anterior cervical disectomy. *European Spine Journal*.

[32] Baron E. M., Soliman A. M. S., Simpson L., Gaughan J. P., Young W. F. (2003). Dysphagia, hoarseness, and unilateral true vocal fold motion impairment following anterior cervical diskectomy and fusion. *The Annals of Otology, Rhinology and Laryngology*.

[33] Sakaura H., Hosono N., Mukai Y., Ishii T., Iwasaki M., Yoshikawa H. (2005). Long-term outcome of laminoplasty for cervical myelopathy due to disc herniation: a comparative study of laminoplasty and anterior spinal fusion. *Spine*.

[34] Kim S., Lee S.-H., Kim E.-S., Eoh W. (2014). Clinical and radiographic analysis of C5 palsy after anterior cervical decompression and fusion for cervical degenerative disease. *Journal of Spinal Disorders & Techniques*.

[35] Galhom A. E. (2013). Comparison between Polyetheretherketone (PEEK) cages versus an iliac-crest autograft used in treatment of single or double level anterior cervical discectomy. *The Medical Journal of Cairo University*.

[36] Kulkarni A. G., Hee H. T., Wong H. K. (2007). Solis cage (PEEK) for anterior cervical fusion: preliminary radiological results with emphasis on fusion and subsidence. *The Spine Journal*.

[37] Cabraja M., Oezdemir S., Koeppen D., Kroppenstedt S. (2012). Anterior cervical discectomy and fusion: comparison of titanium and polyetheretherketone cages. *BMC Musculoskeletal Disorders*.

[38] Song K.-J., Choi B.-W., Jeon T.-S., Lee K.-B., Chang H. (2011). Adjacent segment degenerative disease: is it due to disease progression or a fusion-associated phenomenon? Comparison between segments adjacent to the fused and non-fused segments. *European Spine Journal*.

[39] Teramoto T., Ohmori K., Takatsu T. (1994). Long-term results of the anterior cervical spondylodesis. *Neurosurgery*.

[40] Herkowitz H. N., Kurz L. T., Overholt D. P. (1990). Surgical management of cervical soft disc herniation: a comparison between the anterior and posterior approach. *Spine*.

[41] Williams J. L., Allen M. B., Harkess J. W. (1968). Late results of cervical discectomy and interbody fusion: some factors influencing the results. *The Journal of Bone and Joint Surgery—Series A*.

[42] Ha S.-K., Park J.-Y., Kim S.-H., Lim D.-J., Kim S.-D., Lee S.-K. (2008). Radiologic assessment of subsidence in stand-alone cervical polyetheretherketone (PEEK) cage. *Journal of Korean Neurosurgical Society*.

[43] Song K.-J., Taghavi C. E., Lee K.-B., Song J.-H., Eun J.-P. (2009). The efficacy of plate construct augmentation versus cage alone in anterior cervical fusion. *Spine*.

[44] Kao T.-H., Wu C.-H., Chou Y.-C., Chen H.-T., Chen W.-H., Tsou H.-K. (2014). Risk factors for subsidence in anterior cervical fusion with stand-alone polyetheretherketone (PEEK) cages: a review of 82 cases and 182 levels. *Archives of Orthopaedic and Trauma Surgery*.

[45] Kast E., Derakhshani S., Bothmann M., Oberle J. (2009). Subsidence after anterior cervical inter-body fusion. A randomized prospective clinical trial. *Neurosurgical Review*.

[46] Cho D.-Y., Liau W.-R., Lee W.-Y. (2002). Preliminary experience using a polyetheretherketone (PEEK) cage in the treatment of cervical disc disease. *Neurosurgery*.

[47] Niu C.-C., Liao J.-C., Chen W.-J., Chen L.-H. (2010). Outcomes of interbody fusion cages used in 1 and 2-levels anterior cervical discectomy and fusion: titanium cages versus polyetheretherketone (PEEK) cages. *Journal of Spinal Disorders and Techniques*.

